# Community Landscape of Aging and Homelessness in Denver: A Qualitative Study

**DOI:** 10.1093/geroni/igaf122.4355

**Published:** 2025-12-31

**Authors:** Pilar Ingle, Riley Coyne, Myra Nagy, John Olander, Ingrid Backes, Johnna Flood, Liane Morrison, Asia Cutforth

**Affiliations:** University of Denver, Denver, Colorado, United States; University of Denver, Denver, Colorado, United States; Elevated Denver, Denver, Colorado, United States; Elevated Denver, Denver, Colorado, United States; University of Denver, Denver, Colorado, United States; Elevated Denver, Denver, Colorado, United States; Elevated Denver, Denver, Colorado, United States; University of Denver, University of Denver, Colorado, United States

## Abstract

Older adults (50+) represent the largest age cohort of single adults experiencing homelessness in the U.S. However, they are largely unrecognized by policy makers, and many homeless service organizations are unequipped to appropriately serve their aging clients. This community-engaged qualitative project centers the perspectives of unhoused older adults to identify strengths and gaps in services to inform opportunities for meaningful change. Twenty individuals (ages 61-77, 11 females, 9 males) currently or recently unhoused were recruited to participate in semi-structured interviews from two Denver, CO nonprofits dedicated to serving older unhoused clients. An inductive thematic analysis approach was used to code interviews and identify themes. Interviews lasted an average of 43 minutes. The following themes were identified: 1) unaffordable housing costs contributed to loss of housing, despite most participants having worked most of their lives; 2) overnight shelters, while providing necessary services, feel unsafe and often do not help individuals move forward; 3) aging-specific homeless service organizations were supportive for enrolling in benefits and finding housing, yet needed healthcare services were difficult to access for many; and 4) community and sense of belonging are critical, along with a willingness to ask for help. Meaningful inclusion of older adults who have experienced homelessness is essential for research and program/policy development to ensure relevant, appropriate services and reforms. Our findings further validate systemic failures in supporting our elders’ stability and opportunities to thrive in later life, and underscore the need for homelessness and healthcare services dedicated to unhoused older adults.

